# Role of thromboelastography in the evaluation of septic shock patients with normal prothrombin time and activated partial thromboplastin time

**DOI:** 10.1038/s41598-021-91221-3

**Published:** 2021-06-04

**Authors:** Sang-Min Kim, Sang-Il Kim, Gina Yu, June-Sung Kim, Seok In Hong, Bora Chae, Yo Sep Shin, Youn-Jung Kim, Seongsoo Jang, Won Young Kim

**Affiliations:** 1 Department of Emergency Medicine, University of Ulsan College of Medicine, Asan Medical Center, 88, Olimpic-ro 43-gil, Songpa-gu, Seoul, 05505 Korea; 2 Department of Emergency Medicine, Soonchunhyang University Seoul Hospital, Seoul, Korea; 3 Department of Emergency Medicine, Gangnam Severance Hospital, Seoul, Korea; 4 Department of Laboratory Medicine, University of Ulsan College of Medicine, Asan Medical Center, Seoul, Korea

**Keywords:** Medical research, Diagnostic markers

## Abstract

Coagulopathy is frequent in septic shock and plays a key role in multiple organ dysfunction. The aim of this study is to investigate application values of thromboelastography (TEG) for outcome in septic shock patients with a normal value of prothrombin time (PT) and active partial thromboplastin time (aPTT). Prospective observational study using 1298 consecutive septic shock patients with TEG at admission was conducted at the emergency department (ED) of a tertiary care hospital in South Korea between 2016 and 2019. After excluding overt-disseminated intravascular coagulation (DIC) defined by scoring system, we included patients with a normal value of international normalized ratio ≤ 1.3 and aPTT ≤ 34 s. The primary outcome was 28-day mortality. 893 patients were included and 129 patients with overt DIC were excluded. Of the 764 remaining patients, 414 (54.2%) patients showed normal PT and aPTT (28-day mortality rate, 11.4%). TEG values such as reaction time, kinetic time (K), alpha angle (α), maximum amplitude (MA) and lysis index (LY 30) showed no significant mean difference between the survivor and non-survivor groups. However, hypocoagulable TEG values such as α < 53° (12.0% vs. 23.4%; p = 0.039), and MA < 50 mm (6.3% vs. 21.3%; p = 0.002) were significantly higher in the non-survived group. In multivariate analysis, hypocoagulable state (defined as K > 3 and α < 53 and MA < 50) was independent factors associated with increased risk of death (OR 4.882 [95% CI, 1.698–14.035]; p = 0.003). In conclusion, septic shock patients with normal PT and aPTT can be associated with impaired TEG profile, such as hypocoagulability, associated with increased mortality.

## Introduction

Septic shock patients frequently exhibit coagulation abnormalities, which play a major role in multiorgan dysfunction^1^. The disturbance in the coagulation process varies with the increasing disease severity and ranges from normal to hypercoagulability, hyperfibrinolysis, and ultimately hypocoagulability^2–4^. Sepsis patients have a complex coagulation status; hence, timely assessment is essential for the diagnosis of the condition. Conventional coagulation tests, such as prothrombin time (PT) and activated partial thromboplastin time (aPTT), are commonly used for evaluating the coagulation status. However, the aforementioned tests do not evaluate the entire coagulation process and their accuracy in detecting in vivo hypocoagulability is questionable^5^. Moreover, previous studies have reported that conventional coagulation tests have limited accuracy with regard to characterization of hemostatic profiles and predicting the risk associated with bleeding in critically ill patients^6,7^.

**Figure 1:**
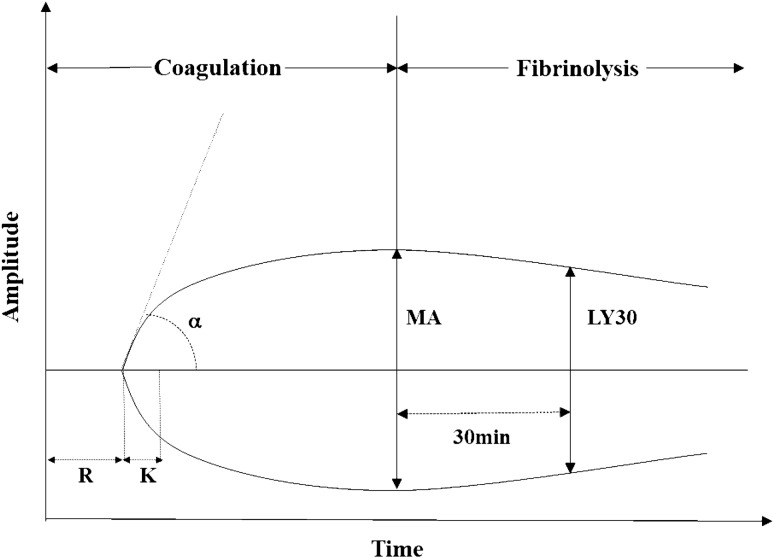
Diagram of TEG. *TEG* thromboelastography; *R* reaction time; *K* kinetic time; *MA* maximal amplitude; *α* alpha angle; *LY30* percentage of lysis after 30 min.

Thromboelastography (TEG) is a point-of-care test that evaluates the entire process of clot formation and dissolution. The major parameters of TEG are reaction time (R), which is the time period from the initiation of the test until the beginning of clot formation, and K-time (K) and alpha angle (α), which reflect the growth kinetics of the clot. Clot strength is represented by the maximum amplitude (MA) of the trace. The degree of fibrinolysis is reflected by the difference between MA and the amplitude measured after 30 min (Fig. 1)^8,9^. Previous studies have reported that TEG is useful in the diagnosis of coagulation abnormalities in sepsis patients and may be associated with the clinical prognosis^10,11^. However, these reports are limited by their small sample size. In addition, the role of TEG in septic shock patients with normal coagulation profiles has been sufficiently studied.

We hypothesized that TEG might provide additional information regarding coagulation dysfunction associated with mortality, even in septic shock patients with normal PT and aPTT. We compared the TEG values between the survivor and non-survivor groups to investigate the clinical implications of TEG in septic shock patients with normal coagulation profiles.

## Results

**Figure 2:**
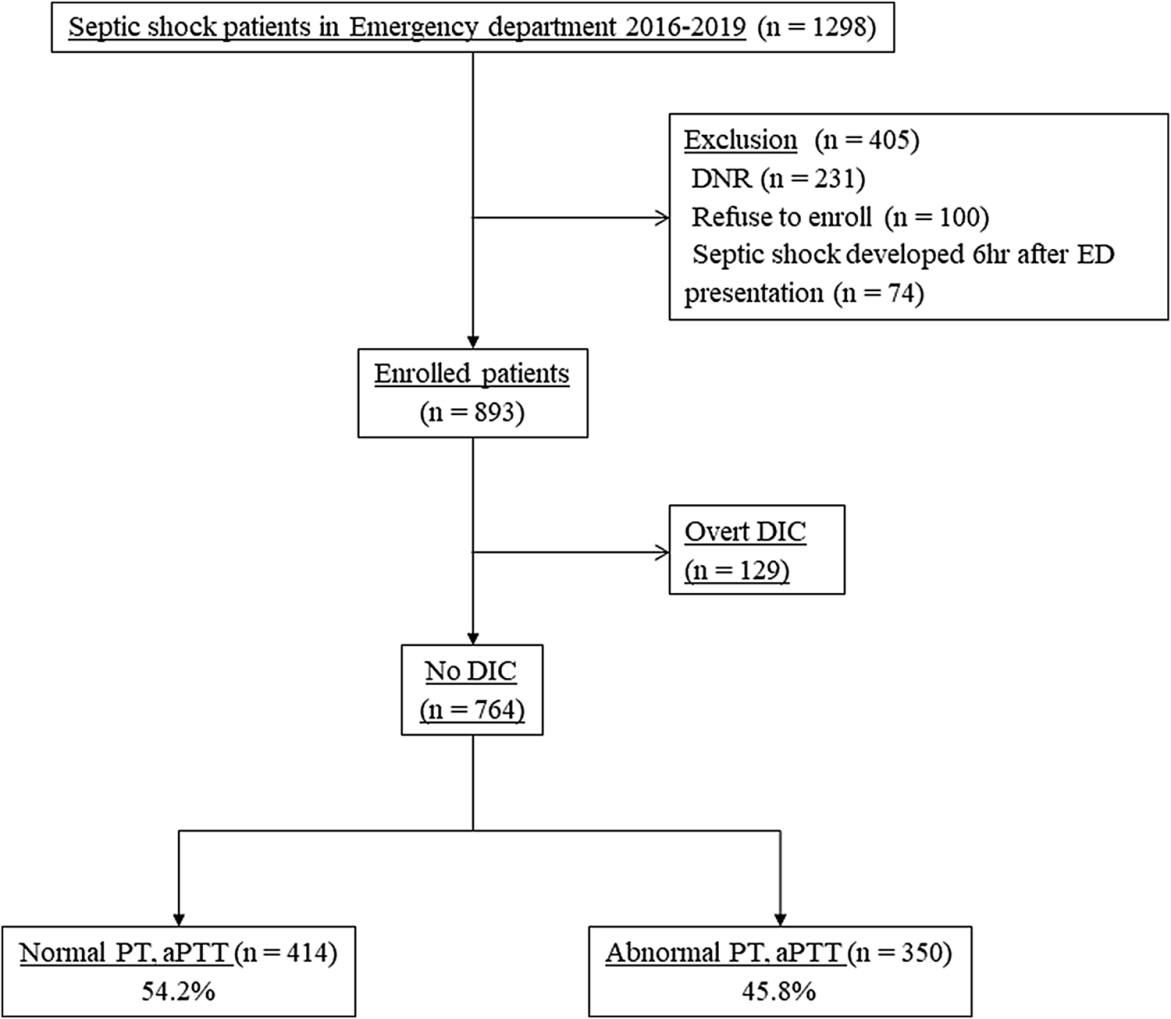
Flow diagram of the study subjects. *TEG* thromboelastography; *DNR* do not resuscitate; *ED* emergency department; *DIC* disseminated intravascular coagulation; *PT* prothrombin time; *aPTT* activated partial thromboplastin time.

We analyzed the data pertaining to 1298 patients who underwent TEG at the time of admission to the ED. Of these patients, 231 patients who were under “do-not-attempt-resuscitation” orders, 100 patients who refused to participate in the study, and 74 patients who developed septic shock 6 h after reporting to the ED were excluded. Consequently, we included 893 septic shock patients. We excluded 129 patients with overt disseminated intravascular coagulation (DIC) defined as an International Society on Thrombosis and Hemostatis (ISTH) score ≥ 5 points. Among the remaining study subjects, 414 (54.2%) patients had normal PT and aPTT results, and their 28-day mortality rate was 11.4% (Fig. 2).

The baseline characteristics, including demographic, clinical, and laboratory data pertaining to the survivor and non-survivor groups, are presented in Table 1. There was no significant difference between the two groups with regard to age, sex, and medical history. Pulmonary infection was more frequently observed in the non-survivor group than in the survivor group (22.1% versus 51.1%; p < 0.001), whereas genitourinary (18.8% versus 6.4%; p = 0.039) and hepatobiliary (32.7% versus 17.0%; p = 0.029) infections were more frequently observed in the survivor group than in the non-survivor group. There was no statistically significant difference between the two groups with regard to the initial laboratory data, except albumin levels (2.9 g/dL [interquartile range (IQR), 2.5–3.4] versus 2.6 g/dL [IQR, 2.2–3.1]; p < 0.001) and lactate (2.3 mmol/L [IQR, 1.7–4.2 mmol/L] versus 4.2 mmol/L [IQR, 2.2–7.0]; p < 0.001). The initial SOFA score (5 [IQR, 4–7] versus 4 [IQR, 3–6]; p = 0.008) and APACHE II score (20 [IQR, 14–25] versus 14 [IQR, 10–19]; p < 0.001) were significantly higher in the non-survivor group than in the survivor group.

**Table 1:** Baseline characteristics of the study population.

Characteristics	All patients n = 414	Survivors n = 367	Non-survivors n = 47	p-value
Age, years	66.2 ± 12.4	65.8 ± 12.3	69.4 ± 12.9	0.064
Male	238 (57.5)	210 (57.2)	28 (59.6)	0.876
**Medical history**				
Hypertension	147 (35.5)	132 (36.0)	15 (31.9)	0.631
Diabetes mellitus	106 (25.6)	90 (24.5)	16 (34.0)	0.160
Malignancy	196 (47.3)	169 (46.0)	27 (57.4)	0.163
**Source of infection**				
Pulmonary	105 (25.4)	81 (22.1)	24 (51.1)	0.000
Genitourinary	72 (17.4)	69 (18.8)	3 (6.4)	0.039
Hepatobiliary	128 (30.9)	120 (32.7)	8 (17.0)	0.029
Others	15 (3.6)	13 (3.5)	2 (4.3)	0.683
**Laboratory findings**				
White blood cell, × 10^3^/uL	9.0 [5.3–14.8]	8.8 [5.3–14.3]	11.3 [5.9–17.7]	0.143
Hemoglobin, g/dL	11.0 [9.5–12.7]	11.0 [9.7–12.7]	11.3 [8.9–13.3]	0.902
Creatinine, mg/dL	1.2 [0.9–1.8]	1.2 [0.9–1.7]	1.3 [0.8–2.2]	0.567
Albumin, g/dL	2.9 [2.4–3.3]	2.9 [2.5–3.4]	2.6 [2.2–3.1]	0.010
Total bilirubin, mg/dL	0.9 [0.6–1.7]	0.9 [0.6–1.7]	0.8 [0.5–1.8]	0.126
CRP, mg/dL	7.7 [3.8–18.3]	7.5 [3.7–18.3]	9.0 [4.3–19.3]	0.361
Initial Lactate, mmol/L	2.5 [1.7–4.5]	2.3 [1.7–4.2]	4.1 [2.2–7.0]	0.000
Initial SOFA score	5.0 [3.0–6.0]	4.0 [3.0–6.0]	5.0 [4.0–7.0]	0.008
APACHE II score	14 [10–19]	14 [10–19]	20 [14–25]	0.000

Values are expressed as the mean ± standard deviation, median [interquartile range], or number (%).

*CRP* C-reactive protein; *SOFA* Sequential Organ Failure Assessment; *APACHE* Acute Physiology and Chronic Health Evaluation.

The coagulation profiles of subjects in the survivor and non-survivor groups are shown in Table 2. There was no significant difference between the groups with regard to platelet count, PT (INR), aPTT, D-dimer level, fibrinogen degradation product (FDP) level, and fibrinogen level. Among the study subjects, two (0.5%) patients had fibrinogen levels < 100 mg/dL and significant difference was observed between the two groups (0.0% versus 4.3%; p = 0.012) with regard to the same. There was no significant difference between the groups with regard to platelet count < 100,000 (18.5% versus 25.5%; p = 0.245).

**Table 2:** Comparison of the coagulation profile in each group.

Variables	All patients n = 414	Survivor n = 367	Non-survivor n = 47	p-value
Platelet, × 10^3^/uL	171 [123–246]	168 [127–242]	200 [99–303]	0.432
PT (INR)	1.16 [1.09–1.22]	1.15 [1.09–1.22]	1.17 [1.11–1.25]	0.171
aPTT, s	28.0 [25.0–30.5]	28.0 [25.1–30.5]	28.3 [24.6–31.5]	0.743
D-dimer, ug/mL	3.6 [1.7–7.3]	3.6 [1.7–7.2]	4.9 [2.6–7.6]	0.198
FDP, ug/mL	12.0 [6.4–21.1]	11.4 [6.3–20.8]	15.5 [8.8–25.5]	0.166
Fibrinogen, mg/dL	401 [314–549]	398 [314–547]	430 [327–595]	0.491
Fibrinogen < 100 mg/dL	2 (0.5%)	0 (0.0%)	2 (4.3%)	0.012
Platelet < 100, × 10^3^/uL	80 (19.3%)	68 (18.5%)	12 (25.5%)	0.245

Values are expressed as the median [interquartile range] or number (%).

*PT* prothrombin time; *INR* international normalized ratio; *aPTT* activated partial thromboplastin time; *FDP* fibrinogen degradation production; *ISTH* International Society on Thrombosis and Hemostasis; *DIC* disseminated intravascular coagulation.

**Figure 3:**
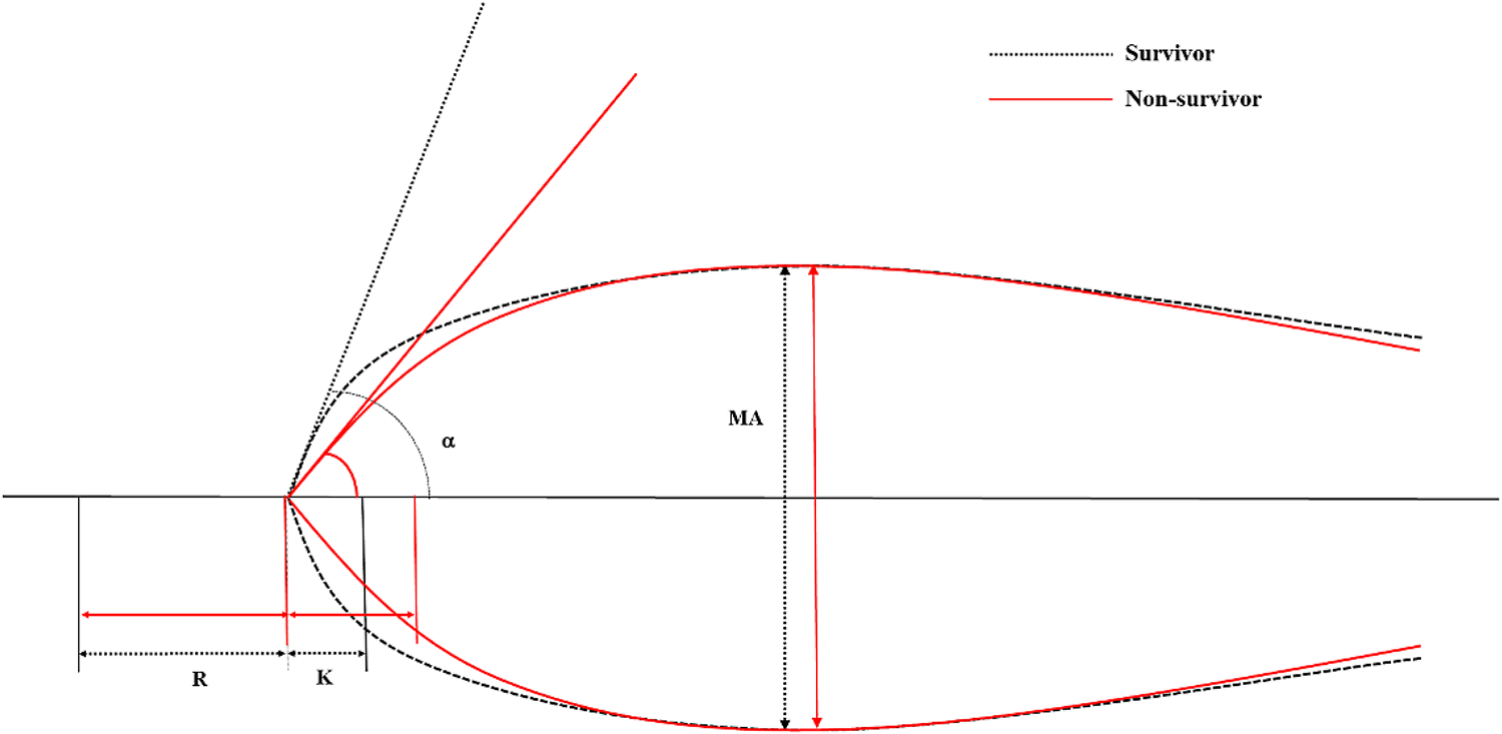
Graphical representation of thromboelastographic tracings and parameters pertaining to the subjects in the survivor (Black dotted line) and non-survivor (Red solid line) groups. *R* reaction time; *K* kinetic time; *α* alpha angle; *MA* maximal amplitude.

TEG parameters pertaining to each group are presented in Table 3 and Fig. 3. The non-survivor group showed slightly prolonged K (1.7 [IQR, 1.1–2.3] versus 1.4 [IQR, 1.1–1.9]; p = 0.131) and decreased α (66.0 [IQR, 55.6–74.2] versus 68.8 [IQR, 61.2–73.5]; p = 0.148) compared to the survivor group. However, the difference was not statistically significant. There was no significant difference between the two with regard to R, MA, and LY30.

**Table 3:** Comparison of the TEG parameters in each group.

Variables	All patients n = 414	Survivors n = 367	Non-survivors n = 47	p-value
R, min	4.9 [3.8–6.0]	4.9 [3.8–5.9]	4.9 [4.1–6.6]	0.225
K, min	1.4 [1.1–1.9]	1.4 [1.1–1.9]	1.7 [1.1–2.3]	0.131
Alpha angle, degree	68.4 [60.8–73.5]	68.8 [61.2–73.5]	66.0 [55.6–74.2]	0.148
MA, mm	66.9 [59.9–72.8]	66.8 [60.1–72.5]	67.6 [50.7–74.3]	0.540
LY30, %	0.0 [0.0–0.7]	0.1 [0.0–0.7]	0.0 [0.0–0.4]	0.062
R > 10.0, min	27 (6.5%)	22 (6.0%)	5 (10.6%)	0.214
K > 3.0, min	40 (9.7%)	32 (8.7%)	8 (17.0%)	0.109
Angle < 53, degree	55 (13.3%)	44 (12.0%)	11 (23.4%)	0.039
MA < 50, mm	33 (8.0%)	23 (6.3%)	10 (21.3%)	0.002
K > 3.0, Angle < 53, and MA < 50	20 (4.8%)	13 (3.5%)	7 (14.9%)	0.004

Values are expressed as the median [interquartile range] or number (%).

*AUC* area under the curve; *TEG* thromboelastography.

An analysis of the hypocoagulable state (R > 10 min, K > 3 min, α < 53°, and MA < 50 mm) showed that there were no significant difference between the groups with regard to K > 3 min (8.7% versus 17.0%; p = 0.109) and R > 10 min (6.0% vs. 10.6%; p = 0.214). However, patients in non-survivor group more frequently had α < 53° (23.4% versus 12.0%; p = 0.039) and MA < 50 mm (21.3% versus 6.3%; p = 0.002) than those in the survivor group. The difference was statistically significant. More patients in the non-survivor group had a hypocoagulable state group (K > 3 min, α < 53°, MA < 50 mm) than those in the survivor group (14.9% versus 3.5%; p = 0.004). The difference was statistically significant.

A univariate analysis was performed with potential confounding factors (age, male sex, initial lactate levels, SOFA score, APACHE II score), hypocoagulable state defined by TEG (R > 10 min, K > 3 min, α < 53°, MA < 50 mm), and the subgroup that represented the hypocoagulable state (K > 3 min, α < 53°, MA < 50 mm). In multivariate logistic regression analysis, initial lactate levels (odds ratio [OR], 1.220 [95% confidence interval (CI), 1.095–1.359]), APACHE II score (OR 1.065 [95% CI, 1.024–1.109], and hypocoagulable state (OR 4.882 [95% CI, 1.698–14.035] were independent factors associated with the 28-day mortality rate (Table 4).

**Table 4:** Univariate and multivariate analysis for predicting the outcomes in septic shock patients.

Characteristics	Univariate OR [95% CI]	Multivariate OR [95% CI]	p-value
Age	OR 1.025 [95% CI, 0.998–1.053]		
Male	OR 1.102 [95% CI, 0.594–2.044]		
Initial lactate	OR 1.259 [95% CI, 1.140–1.391]	OR 1.220 [95% CI, 1.095–1.359]	0.000
SOFA score	OR 1.158 [95% CI, 1.043–1.287]	OR 1.050 [95% CI, 0.928–1.187]	
APACHE II	OR 1.085 [95% CI, 1.044–1.127]	OR 1.065 [95% CI, 1.024–1.109]	0.002
R > 10	OR 1.867 [95% CI, 0.672–5.190]		
K > 3.0	OR 2.147 [95% CI, 0.925–4.988]		
Angle < 53	OR 2.243 [95% CI, 1.065–4.725]		
MA < 50	OR 4.042 [95% CI, 1.787–9.143]		
K > 3.0, Angle < 53, or MA < 50	OR 2.216 [95% CI, 1.099–4.469]		
K > 3.0, Angle < 53, and MA < 50	OR 4.765 [95% CI, 1.797–12.637]	OR 4.882 [95% CI, 1.698–14.035]	0.003

Multivariate analysis included logistic regression analysis and backward elimination.

*OR* odds ratio; *CI* confidential interval; *SOFA* Sequential Organ Failure Assessment; *APACHE* Acute Physiology and Chronic Health Evaluation.

## Discussion

In this study, the TEG profiles, such as α < 53° (12.0% versus 23.4%; p = 0.039), and MA < 50 mm (6.3% versus 21.3%; p = 0.002), were significantly lower in septic shock patients with normal PT and aPTT in the survivor group than in those in the non-survivor group. Multivariate analysis revealed that a hypocoagulable state, defined by K > 3.0 min, α < 53°, and MA < 50 mm, was an independent risk factor associated with the increased risk of mortality (OR 4.882 [95% CI, 1.698–14.035]; p = 0.003).

To the best of our knowledge, our study is the first to investigate the clinical implications of TEG in sepsis patients with normal PT and aPTT. Further, our study included a large sample size involving septic shock patients who underwent TEG at the time of presentation to the ED.

TEG profiles, such as α < 53° and MA < 50 mm, were significantly higher in the non-survivor group than in the survivor group. MA and α are factors associated with the function of fibrinogen, which is a critical component and substrate in the process of clot formation, amplification, and strength^12^. Nonetheless, we did not observe any significant mean difference between the survivor and non-survivor groups with regard to the fibrinogen levels, which is intriguing. Moreover, the analysis of the patients with fibrinogen levels < 100 mg/dL included only two (0.5%) patients. Previous reports have stated that the fibrinogen level remains at falsely normal or even higher levels up until the late stages of disease progression^13,14^; hence, fibrinogen levels may not reflect the actual condition of the patients.

In TEG, the value of MA reflects the function of the platelet. Our study showed that patients with MA < 50 mm were more frequent in the non-survivor group. It suggests that the function of platelet is decreased in the non-survivor group. However, absolute platelet count and the proportion of thrombocytopenia did not significantly differ in the survivor and non-survivor groups. It is reported that platelet function is decreased in sepsis which could be caused by decreased agonist-induced platelet aggregation and dysregulated activators of platelet aggregation^15^. Furthermore, Adamzik et al. reported that marked differences in platelet function were observed in survivors and non-survivors of severe sepsis^16^. Our result supports the previous study.

Hypocoagulable state, defined by the TEG profile (K > 3 min, α < 53°, MA < 50 mm), was independently associated with an increased mortality rate with a more than five-fold higher OR. In sepsis, the activation of coagulation towards hypercoagulability was observed in the early phase of coagulopathy^17^. However, Ostrowski et al. performed a study that involved severe sepsis patients who were admitted to the ICU and reported that hypocoagulability at the time of admission was associated with an increased mortality rate with an OR of 4.2^10^. Another study showed that progressive hypocoagulability, defined by TEG variables, was significantly associated with the mortality rate^11^. This could be explained by the difference of study population that our study only included septic shock which is not in the early phase of disease. So, hypocoagulability, which may not be expected in the normal value of PT and aPTT, was more frequently observed in non-survivors. However, observed hypocoagulability does not suggest that patients have a bleeding tendency. In sepsis-associated DIC, organ dysfunction is more frequent than bleeding, which is a frequent complication of DIC with the hematologic disorder^18^. So, it might be a precise interpretation that impaired TEG profile such as hypocoagulability was associated with more progressed disease status with organ dysfunction.

The protein C anticoagulant pathway serves as a major system for controlling thrombosis, limiting inflammatory responses in response to cytokines and ischemia^19^. Thrombomodulin and EPCR are essential components of the pathway which augments protein C activation. In sepsis, this system is compromised, resulting in impaired fibrinolysis and associated with increased mortality^4,20^. In our study, LY30 was low in both the survivor and non-survivor groups, suggesting impaired fibrinolysis. However, there were no significant differences in LY30 between the groups as conventional TEG cannot easily detect hypofibrinolysis. Panigada et al., which investigated fibrinolysis in sepsis patients with Urokinase Modified TEG, showed that LY30 was lower in the patients with more severe organ dysfunction^21^. If such a method is applied, it would be helpful to compare the fibrinolysis between the groups.

We did not observe any significant difference in the coagulation profiles between the survivor and non-survivor groups. However, the TEG profiles were significantly different between the groups. The aforementioned results are concurrent with the results reported by a previous study, which evaluated coagulopathy in patients admitted at the time of ICU admission. Johansson et al. reported that the results of a conventional coagulation analysis, including aPTT and INR, did not show any difference between the survivor and non-survivor groups^22^. This suggests that hidden coagulopathy could progress in sepsis patients prior to the change in the conventional coagulation profiles, even after the presentation of shock symptoms. Our study results support the finding that TEG can identify sepsis patients with an increased risk of mortality, even if the coagulation profiles such as PT and aPTT are normal. Our study also suggests that further research with relatively a larger sample size is needed to confirm the study result regarding the hypocoagulable state. Although our showed that TEG could define the coagulation status in patients with normal coagulation profiles, the results should be interpreted with caution. Our study does not imply that TEG is superior to other routine coagulation tests or could replace the same; however, it suggests that TEG may provide additional, clinically relevant information that can predict the clinical outcomes or the probability of developing coagulopathy. Moreover, large, multicenter trials are required to confirm the role of TEG in sepsis patients with normal coagulation profiles.

Our study has several limitations. First, this study was a single-center study; hence, the results may not necessarily reflect the general population, regardless of the large sample size. Second, many patients were excluded owing to the “do-not-resuscitate” status. As most of the “do-not-resuscitate” orders were affected because the patients could not recover from the disease, it could lessen the severity of the condition under investigation. Furthermore, the study was conducted at a tertiary hospital; hence, the condition of the patients transferred from other hospitals would be more severe than that of patients who directly report to the ED. Third, we only included patients with normal PT and aPTT. It would be ideal to investigate the role of TEG in normal coagulation profile to exclude all the patients with low antithrombin, high D-dimer, and low fibrinogen. However, those tests provide important information about coagulation status, which is not routinely performed. Considering the advantage of TEG that it can be performed at bedside, it could be appropriate to compare routine coagulation tests such as PT and aPTT. However, if another definition is used, the results would be different. Fourth, the reference range provided by the manufacturer is relatively broad and does not consider the age or sex differences. In addition, previous studies have reported that TEG can be used for monitoring changes associated with hemostasis within an individual rather than as a diagnostic “snapshot” related to a general reference range^23,24^. Consequently, the pre-TEG values in septic shock patients can help detect changes in an individual and could provide crucial information. Fifth, the timing of TEG was not controlled and lacked uniformity. Although we excluded patients who had developed septic shock 6 h after presentation, prompt recognition of the condition is challenging owing to the varied manifestations of septic shock. Future prospective, multicenter studies should be conducted to elucidate the efficacy of TEG for detection of hidden coagulopathy in septic shock patients.

In conclusion, septic shock patients with normal PT and aPTT can be associated with impaired TEG profile, such as hypocoagulable state, associated with increased mortality. It implies that TEG could provide additional, clinically relevant information, even if the coagulation profiles such as PT and aPTT are normal.

## Materials and methods

### Study design and population

This prospective, observational, single-center, registry-based study was conducted in the emergency department (ED) of a tertiary referral academic center in Seoul, South Korea, which provides treatment to approximately 120,000 patients per annum. This study included all adult patients aged ≥ 18 years with septic shock who underwent TEG at the time of admission to the ED from January 2016 to December 2019^25^. The study was approved by the Institutional Review Board of the Asan Medical Center (Study No. 2016-0548), and the requirement for informed consent was waived because this study involved the analysis of case registry records. All procedures performed in this study were in accordance with the ethical standards of the institutional research committee and with the 1964 Helsinki Declaration and its later amendments or comparable ethical standards.

Septic shock was defined as refractory hypotension (systolic blood pressure < 90 mmHg and mean arterial pressure < 70 mmHg) that warranted administration of vasopressor agents despite administration of a minimum of 20–30 mL/kg of intravenous crystalloids or hypoperfusion (blood lactate concentration ≥ 4 mmol/L) with suspected or confirmed infection, in accordance with the definitions reported in previous literature^26^. Septic shock patients who were under “do-not-attempt-resuscitation” orders, those who refused to participate in the study, or those who developed septic shock 6 h after presenting to the ED were excluded from the study. We excluded patients with overt-DIC defined by the criteria outlined by the ISTH, i.e., and ISTH DIC score ≥ 5^27^. We included patients with a normal value of international normalized ratio (INR) ≤ 1.3 and aPTT ≤ 34 s.

### Data collection

Demographic and clinical data on age, sex, past medical history, and source of infection were obtained from the electronic medical records of registry-enrolled patients. Blood samples were obtained from all patients within 15 min after reporting to the ED. The white blood cell count; levels of hemoglobin, C-reactive protein, procalcitonin, blood urea nitrogen, creatinine, brain natriuretic peptide, and coagulation factors; and results of liver function tests and cardiac enzyme tests were evaluated. After recognition of the onset of the initial stages of septic shock, the Sequential Organ Failure Assessment (SOFA) score and the Acute Physiology And Chronic Health Evaluation II (APACHE II) score were estimated in the ED^28,29^.

### Thromboelastography

All patients underwent TEG after the concurrent detection of septic shock. Whole blood was drawn into vacutainer tubes (volume: 4 mL) containing citrate, which was immediately transferred to the hospital blood bank and TEG analyses were performed by trained personals using standardized procedures, in accordance with the manufacturer’s recommendations using a TEG 5000 Hemostasis Analyzer System (Haemoscope Corporation, Chicago, Illinois, USA). The TEG analyzer system measures the physical properties of the clot and records kinetic changes while the container that encloses the whole blood sample rotates and oscillates. The following variables were recorded: reaction time (R, reference interval: 5–10 min; reflects the rate of initial fibrin formation), speed of clot formation (K, reference interval: 1–3 min; reflects the growth kinetics of the clot), alpha angle (α, reference interval: 53°–72°; reflects the growth kinetics of the clot), maximum amplitude (MA, reference interval: 50–70 mm; reflects the clot strength), and lysis after 30 min (LY30, reference interval 0–7.5%; proportional reduction in amplitude after MA; reflects fibrinolysis).

### Statistical analysis

Continuous variables with normal distribution are presented as mean ± standard deviation, and variables with skewed distribution are represented as median and interquartile range. Categorical variables are presented as mean and percentage. To compare the difference among the variables, Student’s t-test or the Wilcoxon rank-sum test and the chi-square test or Fisher’s exact test were used for continuous and categorical variables, respectively. Multivariate analysis was performed, including the binary TEG variable, which represents the hypocoagulable status, to demonstrate the efficacy of TEG as an independent factor in predicting the 28-day mortality rate in septic shock patients. All statistical analyses were performed using SPSS 21.0 (IBM Corp., Armonk, NY). A P-value of < 0.05 was considered statistically significant.
